# Supplementary material for: Ultra-low and ultra-broad-band nonlinear acoustic metamaterials
Source: Nat Commun. 2017 Nov 3;8:1288. doi: 10.1038/s41467-017-00671-9 (PMC5670230; doi:10.1038/s41467-017-00671-9)
Supplement: Supplementary file 1 — Supplementary Information [file 41467_2017_671_MOESM1_ESM.pdf]

Description of Supplementary Files

File Name: Supplementary Information
Description: Supplementary Figures, Supplementary Notes and Supplementary References.

File Name: Peer Review File

Supplementary note 1. Method for equivalent parameters

As shown in Fig. 2b, the flexural motion of the entire attachment in the meta-cell can be modeled as a torsional oscillating system. To determine the equivalent parameters J_0, J_r and k_T, the finite element method (FEM) is used to calculate the low-frequency responses of the entire attachment, and the displacement of the terminal points are shown in Supplementary Fig. 1. The first resonant and anti-resonant frequencies of this system are f_{T1} = 226 Hz and f_{T2} = 1,424 Hz, respectively. In addition, $\omega_{Ti} = 2\pi f_{Ti}$, i = 1, 2.

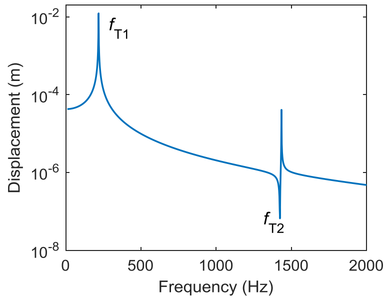

Supplementary Figure 1 | Frequency responses of the rod with concentrated masses. FEM is used to calculate the frequency responses of the entire attachment in Fig. 2b. The parameters of the rod with masses are listed in Table 1. The displacement of the terminal point is shown here. f_{T1} and f_{T2} denote the first resonant and the first anti-resonant frequencies, respectively.

The dynamic inertia of the equivalent torsional oscillators is

$$\bar{J}(\omega) = J_0 + k_T / (\omega_{0T}^2 - \omega^2). \tag{1}$$

The properties of the metamaterial tell us $\bar{J}(\omega_{T1}) = \infty, \bar{J}(\omega_{T2}) = 0$ and thus we derive $k_T / J_r = \omega_{T1}^2$ and $k_T = J_0(\omega_{T2}^2 - \omega_{T1}^2)$. Another relevant equation is the total inertia J_t relative to the fixed point O, $J_t = J_0 + J_r$. For the structure in practice, we have $J_t = \sum_i J_i$, $J_i = m_i \left(r_i^2/4 + d_i^2/12 + d_{oi}^2 \right)$, where m_i, r_i, d_i and d_{oi} are the mass, the radius, the length and the distance between the center of gravity and point O, respectively; and the subscript i denotes the corresponding parameters of the magnets, the strut and the bolt. With the three equations, the equivalent parameters can be solved:

$$k_T = \frac{J_t}{1/(\omega_{T2}^2 - \omega_{T1}^2) + 1/\omega_{T1}^2}, \tag{2}$$

$$J_r = k_T / \omega_{T1}^2, \tag{3}$$

$$J_0 = J_t - J_r. \tag{4}$$

An equivalent system with more Degrees-of-freedoms (DoFs) can describe higher-order motions in the high-frequency range.

Supplementary note 2. Dispersion theory based on FEM

FEM of the nonlinear oscillators

The finite elements of the nonlinear oscillators derive from their motion equations. In case N1, that is, neglecting the influence of the vibro-impact oscillator, the node vector is $\mathbf{v}=[w_0, \theta_0, \bar{w}_r, \theta_r]^T$, where $\bar{w}_r$ denotes the transverse displacement relative to w_0, $\bar{w}_r = w_r - w_0$. Therefore the mass matrix $\mathbf{m}_{er}$, linear stiffness matrix $\mathbf{k}_{erL}$ and the nonlinear stiffness matrix $\mathbf{k}_{erDi}$ of the ith element are

$$\mathbf{m}_{er} = \begin{bmatrix} m_0+m_r & 0 & m_r & 0 \\ 0 & J_0 & 0 & 0 \\ m_r & 0 & m_r & 0 \\ 0 & 0 & 0 & J_r \end{bmatrix}, \tag{5}$$

$$\mathbf{k}_{erL} = \begin{bmatrix} 0 & 0 & 0 & 0 \\ 0 & k_T & 0 & -k_T \\ 0 & 0 & k_1 & 0 \\ 0 & -k_T & 0 & k_T \end{bmatrix}, \tag{6}$$

$$\mathbf{k}_{erDi} = \begin{bmatrix} 0 & & & \\ & 0 & & \\ & & k_2\bar{w}_{ri}^2 & \\ & & & 0 \end{bmatrix}. \tag{7}$$

If we take into consideration of the vibro-impact oscillator, as shown in Fig. 2c, the motion equation of the coupled torsion-vibro-impact system in the NAM beam is

$$J_0\ddot{\theta}_0 = k_T(\theta_r - \theta_0) + M_0(t), \tag{8}$$

$$J_r\ddot{\theta}_r + m_r l_r^2(\ddot{\phi}_r + \ddot{\theta}_r) = -k_T(\theta_r - \theta_0), \tag{9}$$

$$m_r(\ddot{\phi}_r + \ddot{\theta}_r) = -k_3\phi_r - k_c l_r^2 \phi_r^n, \tag{10}$$

where $\phi_r = u_r/l_r - \theta_r$. In this case, by specifying the node vector as $\mathbf{v}=[w_0, \theta_0, \bar{w}_r, \theta_r, \phi_r]^T$, the element matrices are

$$\mathbf{m}_{er} = \begin{bmatrix} m_0+m_r & 0 & m_r & & \\ 0 & J_0 & 0 & & \\ m_r & 0 & m_r & & \\ & & & J_r + m_r l_r^2 & m_r l_r^2 \\ & & & m_r & m_r \end{bmatrix}, \tag{11}$$

$$\mathbf{k}_{erL} = \begin{bmatrix} 0 & 0 & 0 & 0 & 0 \\ 0 & k_T & 0 & -k_T & 0 \\ 0 & 0 & k_1 & 0 & 0 \\ 0 & -k_T & 0 & k_T & 0 \\ 0 & 0 & 0 & 0 & k_3 \end{bmatrix}, \tag{12}$$

$$\mathbf{k}_{\text{erD}i} = \begin{bmatrix} 0 & & & & \\ & 0 & & & \\ & & k_2 \bar{w}_{ri}^2 & & \\ & & & 0 & \\ & & & & k_c l_r^2 \phi_{ri}^{n-1} \end{bmatrix}. \tag{13}$$

For the NAM plate, a similar expression can be derived. This system is named as NAM-N2. In fact, because the relative coordinates $\bar{w}_r$ and ϕ_r are used, the nonlinear stiffness matrix $\mathbf{k}_{\text{erD}i}$ is decoupled.

In the dispersion solutions detailed below, we use the cubic term n=3 to approximate the vibro-impact oscillator, on which occasion we rewrite $\mathbf{k}_{\text{erD}i}$ as $\mathbf{k}_{\text{erD}i} = [\mathbf{n}] \cdot \mathbf{v}^2$, where $[\mathbf{n}]$ is the coefficient matrix consisting of the nonzero elements k_2 and $k_c l_r^2$. The power of a vector $\mathbf{x}$ is defined as $\mathbf{x}^n = \{x_1^n \cdots x_i^n \cdots\}$.

FEM of a periodic cell of the NAM beam

By a standard FEM process, the motion equation of all the nodes in a cell is

$$\tilde{\mathbf{M}}\ddot{\tilde{\mathbf{u}}} + \tilde{\mathbf{K}}\tilde{\mathbf{u}} + \tilde{\mathbf{N}} \cdot \tilde{\mathbf{u}}^3 = \tilde{\mathbf{f}}, \tag{14}$$

where

$$\tilde{\mathbf{u}} = [\mathbf{u}_L^T \ \ \mathbf{u}_I^T \ \ \mathbf{u}_R^T \ \ \mathbf{u}_a^T]^T, \tag{15}$$

$$\tilde{\mathbf{f}} = [\mathbf{f}_L^T \ \ \mathbf{0} \ \ \mathbf{f}_R^T \ \ \mathbf{0}]^T, \tag{16}$$

in which $\tilde{\mathbf{M}}$, $\tilde{\mathbf{K}}$ and $\tilde{\mathbf{N}}$ denote the mass, the stiffness and the nonlinear stiffness coefficient matrices, respectively; $\tilde{\mathbf{u}}$ denotes the displacement vector of all the nodes; $\tilde{\mathbf{f}}$ is the vector of the node forces; the subscripts, L, I, R and a, symbolize the nodes at the left boundary, inside the primary beam, at the right boundary and of the attachments, respectively; $\mathbf{u}_L = [w_1 \ \ \theta_1]^T = [w_0 \ \ \theta_0]^T$, and $\mathbf{u}_a$ depends on if the vibro-impact motion is considered. For NAM-N1, $\mathbf{u}_a = [\bar{w}_r \ \ \theta_r]^T$ and for NAM-N2 $\mathbf{u}_a = [\bar{w}_r \ \ \theta_r \ \ \phi_r]^T$. In fact, there are only two nonzero elements in $\tilde{\mathbf{N}}$ that are corresponding to the relative displacements $\bar{w}_r$ and ϕ_r.

With Bloch theorem in the one-dimensional (1D) periodic beam, the periodic boundary conditions for a cell are expressed as

$$\mathbf{u}_R = e^{-i\kappa a}\mathbf{u}_L, \tag{17}$$

$$\mathbf{f}_R = -e^{-i\kappa a}\mathbf{f}_L, \tag{18}$$

where $\kappa \in [0, 2\pi]$ denotes the wave vector, a is the lattice constant and i^2 =-1. If $\bar{\mathbf{u}} = [\mathbf{u}_L^T \ \ \mathbf{u}_I^T \ \mathbf{u}_a^T]^T$, $\bar{\mathbf{f}} = [\mathbf{f}_L^T \ \ \mathbf{0} \ \ \mathbf{0}]^T$, one gets

$$\tilde{\mathbf{u}} = \mathbf{R}\bar{\mathbf{u}}, \tag{19}$$

in which,

$$\mathbf{R}=\begin{bmatrix} \mathbf{I} & \mathbf{0} & \mathbf{0} \\ \mathbf{0} & \mathbf{I} & \mathbf{0} \\ e^{-i\kappa a}\mathbf{I} & \mathbf{0} & \mathbf{0} \\ \mathbf{0} & \mathbf{0} & \mathbf{I} \end{bmatrix}. \tag{20}$$

With the relationships in (17), we can prove that

$$\mathbf{R}^{\mathrm{H}}\bar{\mathbf{f}}=\mathbf{0}, \tag{21}$$

where the superscript 'H' denotes the conjugate transpose. Because the elements in $\tilde{\mathbf{N}}$ corresponding to $\mathbf{u}_{\mathrm{L}}^{\mathrm{T}}$ and $\mathbf{u}_{\mathrm{I}}^{\mathrm{T}}$ are zero, for the nonlinear part, one obtains $\mathbf{R}^{\mathrm{H}}\tilde{\mathbf{N}}(\mathbf{R}\bar{\mathbf{u}})^3=\mathbf{R}^{\mathrm{H}}\tilde{\mathbf{N}}\mathbf{R}(\bar{\mathbf{u}})^3$. At last, substitute the old coordinates $\tilde{\mathbf{u}}$ with the new vector $\bar{\mathbf{u}}$, one obtains the new motion equation of a cell. It is

$$\bar{\mathbf{M}}\ddot{\bar{\mathbf{u}}}+\bar{\mathbf{K}}\bar{\mathbf{u}}+\bar{\mathbf{N}}\cdot(\bar{\mathbf{u}})^3=0 \tag{22}$$

where $\bar{\mathbf{M}}=\mathbf{R}^{\mathrm{H}}\tilde{\mathbf{M}}\mathbf{R},\ \bar{\mathbf{K}}=\mathbf{R}^{\mathrm{H}}\tilde{\mathbf{K}}\mathbf{R},\ \bar{\mathbf{N}}=\mathbf{R}^{\mathrm{H}}\tilde{\mathbf{N}}\mathbf{R}$.

FEM of a periodic cell of the NAM plate

For the plate, the displacement vector of every node is $\mathbf{u}_j=[w_j\ \ \theta_{xj}\ \ \theta_{yj}]^{\mathrm{T}}$. With a proper transform and a standard FEM procedure, the motion equation of a cell reads

$$\tilde{\mathbf{M}}\{\ddot{\mathbf{q}}\}+\tilde{\mathbf{K}}\{\mathbf{q}\}+\tilde{\mathbf{N}}\{\mathbf{q}^3\}=\{\tilde{\mathbf{f}}\} \tag{23}$$

where the displacement vector of the whole system is

$$\{\mathbf{q}\}=\{\mathbf{q}_{\mathrm{LB}}^{\mathrm{T}},\mathbf{q}_{\mathrm{B}}^{\mathrm{T}},\mathbf{q}_{\mathrm{RB}}^{\mathrm{T}},\mathbf{q}_{\mathrm{L}}^{\mathrm{T}},\mathbf{q}_{\mathrm{R}}^{\mathrm{T}},\mathbf{q}_{\mathrm{LT}}^{\mathrm{T}},\mathbf{q}_{\mathrm{T}}^{\mathrm{T}},\mathbf{q}_{\mathrm{RT}}^{\mathrm{T}},\mathbf{q}_{\mathrm{I}}^{\mathrm{T}},\mathbf{q}_{\mathrm{r}}^{\mathrm{T}}\}^{\mathrm{T}} \tag{24}$$

As shown in Supplementary Fig. 2, $\mathbf{q}_j$ denotes the set of node displacements, for example, $\mathbf{q}_{\mathrm{L}}$ represents the displacement of all the nodes in the left boundary of a cell. $\mathbf{q}_{\mathrm{r}}$=[$\bar{w}_{\mathrm{r}}$, $\theta_{\mathrm{r}x}$, $\theta_{\mathrm{r}y}$]^{T} or $\mathbf{q}_{\mathrm{r}}$=[$\bar{w}_{\mathrm{r}}$, $\theta_{\mathrm{r}x}$, $\theta_{\mathrm{r}y}$, $\phi_{\mathrm{r}x}$, $\phi_{\mathrm{r}y}$]^{T}, which depends on the system N1 or N2. Correspondingly, the node vector of the force is

$$\{\tilde{\mathbf{f}}\}=\{\mathbf{f}_{\mathrm{LB}}^{\mathrm{T}},\mathbf{f}_{\mathrm{B}}^{\mathrm{T}},\mathbf{f}_{\mathrm{RB}}^{\mathrm{T}},\mathbf{f}_{\mathrm{L}}^{\mathrm{T}},\mathbf{f}_{\mathrm{R}}^{\mathrm{T}},\mathbf{f}_{\mathrm{LT}}^{\mathrm{T}},\mathbf{f}_{\mathrm{T}}^{\mathrm{T}},\mathbf{f}_{\mathrm{RT}}^{\mathrm{T}},\mathbf{f}_{\mathrm{I}}^{\mathrm{T}},\mathbf{f}_{\mathrm{r}}^{\mathrm{T}}\}^{\mathrm{T}} \tag{25}$$

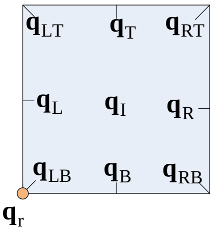

Supplementary Figure 2 | Displacement vectors in a cell of the NAM plate. **q** denotes the displacement vector. The subscripts represent the different positions of the nodes, as labeled near the corresponding position. L: left; B: bottom; R: right; T: top; I: inner; r: the resonator. For example, $\mathbf{q}_{\mathrm{RB}}$ ($\mathbf{q}_{\mathrm{L}}$) means it is the displacement vector of the right bottom corner (the nodes along left boundary apart from

the corners); $\mathbf{q}_\mathrm{I}$ means the displacement vector of all the nodes inside the cell, apart from all the boundaries and corners.

The Bloch theorem of a periodic cell is expressed as

$$\mathbf{q}_{\mathrm{RB}} = e^{ik_x l_x}\mathbf{q}_{\mathrm{LB}}, \tag{26}$$

$$\mathbf{q}_{\mathrm{R}} = e^{ik_x l_x}\mathbf{q}_{\mathrm{L}}, \tag{27}$$

$$\mathbf{q}_{\mathrm{RT}} = e^{i(k_x l_x + k_y l_y)}\mathbf{q}_{\mathrm{LB}}, \tag{28}$$

$$\mathbf{q}_{\mathrm{LT}} = e^{ik_y l_y}\mathbf{q}_{\mathrm{LB}}, \tag{29}$$

$$\mathbf{q}_{\mathrm{T}} = e^{ik_y l_y}\mathbf{q}_{\mathrm{B}}, \tag{30}$$

and

$$\mathbf{f}_{\mathrm{R}} = -e^{ik_x l_x}\mathbf{f}_{\mathrm{L}}, \tag{31}$$

$$\mathbf{f}_{\mathrm{T}} = -e^{ik_y l_y}\mathbf{f}_{\mathrm{B}}, \tag{32}$$

$$\mathbf{f}_{\mathrm{RT}} = -e^{ik_x l_x}\mathbf{f}_{\mathrm{LT}} - e^{ik_y l_y}\mathbf{f}_{\mathrm{RB}} - e^{i(k_x l_x + k_y l_y)}\mathbf{f}_{\mathrm{LB}}, \tag{33}$$

where l_x and l_y are the lattice constants in x and y directions, respectively; $\mathbf{k}=(k_x, k_y)$ is the wave vector of the first Brillouin zone (see Fig. 2g). For the nodes inside the cell and on the attached resonator, $\mathbf{f}_\mathrm{I} = \mathbf{0},\ \mathbf{f}_\mathrm{r} = \mathbf{0}$. By defining a new vector $\{\mathbf{q}\}_\mathrm{c} = \{\mathbf{q}_{\mathrm{LB}}^\mathrm{T}, \mathbf{q}_{\mathrm{B}}^\mathrm{T}, \mathbf{q}_{\mathrm{L}}^\mathrm{T}, \mathbf{q}_{\mathrm{I}}^\mathrm{T}, \mathbf{q}_{\mathrm{r}}^\mathrm{T}\}^\mathrm{T}$, one yields

$$\{\mathbf{q}\}=\mathbf{R}\{\mathbf{q}\}_\mathrm{c}, \tag{34}$$

where

$$\mathbf{R} = \begin{bmatrix} \mathbf{I}_{\mathrm{LB}} & \mathbf{0} & \mathbf{0} & \mathbf{0} & \mathbf{0} \\ \mathbf{0} & \mathbf{I}_{\mathrm{B}} & \mathbf{0} & \mathbf{0} & \mathbf{0} \\ e^{ik_x l_x}\mathbf{I}_{\mathrm{LB}} & \mathbf{0} & \mathbf{0} & \mathbf{0} & \mathbf{0} \\ \mathbf{0} & \mathbf{0} & \mathbf{I}_{\mathrm{L}} & \mathbf{0} & \mathbf{0} \\ \mathbf{0} & \mathbf{0} & e^{ik_x l_x}\mathbf{I}_{\mathrm{L}} & \mathbf{0} & \mathbf{0} \\ e^{ik_y l_y}\mathbf{I}_{\mathrm{LB}} & \mathbf{0} & \mathbf{0} & \mathbf{0} & \mathbf{0} \\ \mathbf{0} & e^{ik_y l_y}\mathbf{I}_{\mathrm{B}} & \mathbf{0} & \mathbf{0} & \mathbf{0} \\ e^{i(k_x l_x + k_y l_y)}\mathbf{I}_{\mathrm{LB}} & \mathbf{0} & \mathbf{0} & \mathbf{0} & \mathbf{0} \\ \mathbf{0} & \mathbf{0} & \mathbf{0} & \mathbf{I}_{\mathrm{I}} & \mathbf{0} \\ \mathbf{0} & \mathbf{0} & \mathbf{0} & \mathbf{0} & \mathbf{I}_{\mathrm{r}} \end{bmatrix}, \tag{35}$$

in which $\mathbf{I}_j$ denotes the identity matrix whose dimension is equal to the corresponding vector $\mathbf{q}_j$. With the relationships of interaction forces between neighbor cells, we can prove that

$$\mathbf{R}^{\mathrm{H}}\{\tilde{\mathbf{f}}\} = \{\mathbf{0}\}. \tag{36}$$

At last, the motion equation of a cell is transformed to

$$\bar{\mathbf{M}}\{\ddot{\mathbf{q}}\}_c + \bar{\mathbf{K}}\{\mathbf{q}\}_c + \bar{\mathbf{N}} \cdot \{\mathbf{q}\}_c^3 = \mathbf{0}, \tag{37}$$

where $\bar{\mathbf{M}} = \mathbf{R}^H \tilde{\mathbf{M}} \mathbf{R}$, $\bar{\mathbf{K}} = \mathbf{R}^H \tilde{\mathbf{K}} \mathbf{R}$, $\bar{\mathbf{N}} = \mathbf{R}^H \tilde{\mathbf{N}} \mathbf{R}$. Equations (22) and (37) have a same form.

Methods to calculate the dispersion relations

The analytical dispersion solutions of the linearized system are obtained with the eigen function

$$\left| \bar{\mathbf{K}} - \omega^2 \bar{\mathbf{M}} \right| = 0 \tag{38}$$

However, only approximate dispersion solutions can be found for nonlinear metamaterials.

For the weakly nonlinear metamaterial, perturbation approach (PA) is used to solve the dispersion relations, as elaborated in the Refs. [1, 2]. The nonlinear dispersion solution derives from PA is

$$\omega = \omega_0 + \varepsilon\omega_1 + O(\varepsilon^2) = \omega_0 + \frac{3\mathbf{U}_0^H \bar{\mathbf{N}}(\mathbf{U}_0)^3}{8\omega_0 \mathbf{U}_0^H \bar{\mathbf{M}} \mathbf{U}_0} + O(\varepsilon^2), \tag{39}$$

where $\mathbf{U}_0$ is the generalized eigenvector belonging to the eigenfrequency ω_0 of the linearized system, that is, $[\bar{\mathbf{K}} - \omega_0^2 \bar{\mathbf{M}}]\mathbf{U}_0 = \mathbf{0}$; $O(\varepsilon^2)$ denotes high-order small quantities.

In our manuscript, PA can only be used to in the case NAM-N1. For NAM-N2, the nonlinearity in vibro-impact oscillator is so strong that PA would present wrong results.

For the strongly nonlinear acoustic metamaterial, harmonic balance method (HBM) can present the dispersion relationships more accurately. Here the wave solution is assumed as $\mathbf{u}(t)$=$\mathbf{U}\sin\omega t$. Balancing the coefficient of $\sin\omega t$, we obtain the first-order harmonic balance solution

$$[\bar{\mathbf{K}} - \omega^2 \bar{\mathbf{M}}]\mathbf{U} + \frac{3}{4}\bar{\mathbf{N}}(\mathbf{U})^3 = \mathbf{0}. \tag{40}$$

In both PA and HBM, by specifying the initial amplitude U_{01} of u_1, we can solve the dispersion frequency ω and other parameters in $\mathbf{U}$.

In the FEM process, a cell of the primary beam is meshed by three conformal elements with same length. And a 2D NAM plate cell is meshed by 2×2 elements. These meshes are accurate enough for low-frequency studies (lower than 1500 Hz). For NAM beam-N2, analytical solutions can be found for the 11-dimensional (11D) system of algebraic equations in Eq. (40). However, for the 2D NAM plate-N2, the system of equations is 16D. It is a high-dimensional system so that it becomes difficult to find analytical solutions with HBM. Therefore only the dispersion solutions of the NAM

plate-N1 are considered and solved by PA.

Supplementary note 3. Nonlinear vibration of NAM beam

In this section, based on the nonlinear FEM, we take into account of four nonlinear sources in the NAM beams: the inertial nonlinearity, the geometrical nonlinearity, the Duffing oscillator and the vibro-impact oscillator. HBM and Harmonic average approach (HAA) are introduced. By studying the influences of different nonlinear sources, we confirm that the Duffing and the vibro-impact oscillators are the most important nonlinear factors. Moreover, the strong nonlinearity of the vibro-impact oscillator is analyzed with a comparative way. At last, we show the experimental results on a LAM beam.

Nonlinear FEM of the NAM beam

1. Nonlinear FEM of a pure beam

The 2D theory for the geometrically exact beam element is based on the assumption of the plane across section. It was derived by Reissner and is valid for finite deflections, rotations and strains[3, 4]. The associate strain-deflection relations are

$$\varepsilon = (1+u_x)\cos\psi + w_x \sin\psi - 1, \tag{41}$$

$$\gamma = w_x \cos\psi - (1+u_x)\sin\psi, \tag{42}$$

$$\kappa = \psi_x, \tag{43}$$

where ε, γ, κ are the axial strain, the shear strain and the curvature, respectively; u is the displacement in axial direction, w is the deflection and ψ is the rotation, as shown in Supplementary Fig. 3; $(\cdot)_x$=d/dx.

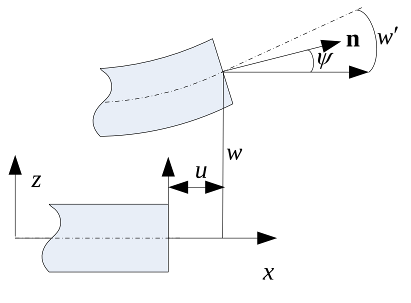

Supplementary Figure 3 | Deformation of the nonlinear beam. For the deformed beam, the node at x generates an axial displacement u, a transverse displacement w and a deflection ψ. The normal vector of the section is **n**. w' denotes the supposed rotation of the middle face[3].

The Eula-Bernoulli beam assumes γ=0. By using this shear constrain, ε is obtained depending upon u and w

$$\varepsilon = \sqrt{(1+u_x)^2 + w_x^2} - 1. \tag{44}$$

By some algebra to solve ψ and then using a derivation, one yields

$$\kappa = \frac{w_{xx}(1+u_x) - u_{xx}w_x}{(1+u_x)^2 + w_x{}^2}. \tag{45}$$

For the free-end beam, the axial deformation can be neglect, that is, ε=0. Using this constrain to Eq. (44) yields

$$u_x = \sqrt{1-w_x^2} - 1 \tag{46}$$

Therefore $u_x \leq 0$, which indicates the beam generates shorten inertial effect. Through an integral, we obtain the displacement induced by the shorten effect at point x

$$u = \int_0^x (\sqrt{1-w_x^2} - 1)dx \tag{47}$$

and

$$u_t = -\int_0^x w_x w_{xt} \Big/ \sqrt{1-w_x^2}\, dx\,. \tag{48}$$

With the Taylor series $1/\sqrt{1-w_x^2} \approx 1 + \frac{1}{2}w_x^2 + \cdots$, one yields

$$u_t = -\int_0^x (w_x w_{xt} + \frac{1}{2}w_x^3 w_{xt} + ...)dx \approx -\int_0^x w_x w_{xt} dx. \tag{49}$$

By inserting u_x into (45), the curvature is obtained

$$\kappa = w_{xx} \Big/ \sqrt{1-w_x^2}\,. \tag{50}$$

Using that Taylor series, we obtain

$$\kappa^2 = w_{xx}^2 + w_x^2 w_{xx}^2 \tag{51}$$

Based on these displacement and stain expressions, the kinetic energy T and strain energy U of an element within length s are

$$T = \frac{1}{2}\int_0^s \rho u_t^2 dx + \frac{1}{2}\int_0^s \rho w_t^2 dx = \frac{1}{2}\int_0^s \rho w_t^2 dx + \frac{1}{2}\int_0^s \rho \left[\int_0^x w_x w_{xt} dx\right]^2 dx, \tag{52}$$

$$U = \frac{1}{2}\int_0^s EA\varepsilon^2 dx + \frac{1}{2}\int_0^s EI\kappa^2 dx = \frac{1}{2}\int_0^s EI(w_{xx}^2 + w_x^2 w_{xx}^2)dx, \tag{53}$$

where $(\cdot)_x$=d/dx and ρ is the mass of unit length. The second term in T denotes the inertial nonlinearity and the second term in U represents the geometrical nonlinearity. In this expression, the geometrical nonlinearity depends both on the transverse strain and the curvature.

At present, most studies on the nonlinear beams use the Galerkin method by assuming that the motion depends on the superposition of several linear modals. This method reduces much DoFs in analyzing for convenience. However, this

method is proper for simple cases. For the nonlinear metamaterial beam coupled with many nonlinear oscillators, modal superposition is improper for broadband analyses. Here, we adopt the nonlinear FEM. In general, we can use the expression of the linear FEM to approximate the displacement field in nonlinear FEM, that is,

$$w(x,t) = \mathbf{N}(x)\boldsymbol{\eta}(t) \tag{54}$$

where $\boldsymbol{\eta}(t)$ is the nodal vector, $\boldsymbol{\eta}(t) = [w_1 \quad \theta_1 \quad w_2 \quad \theta_2]^{\mathrm{T}}$, and $\mathbf{N}(x)$ is the vector of shape functions. If we adopt the finite element coordinate $x=l\xi$, and length of an element is $2l$, the shape function of the conformal element is

$$\mathbf{N}(\xi) = [W_1 \quad W_2 \quad W_3 \quad W_4], \tag{55}$$

where $W_1 = \left(2-3\xi+\xi^3\right)/4$, $W_2 = l\left(1-\xi-\xi^2+\xi^3\right)/4$, $W_3 = \left(2+3\xi-\xi^3\right)/4$ and $W_4 = l\left(-1-\xi+\xi^2+\xi^3\right)/4$.

By inserting $w(x,t)$ in the expressions of T and U, the elemental matrices can be set up with the Lagrange equations. The linear mass matrix $\mathbf{m}_{\mathrm{eL}}$ and the nonlinear inertia matrix $\mathbf{m}_{\mathrm{eI}i}$ for a pure beam element are

$$\mathbf{m}_{\mathrm{eL}} = \rho l \int_{-1}^{1} \mathbf{N}^{\mathrm{T}}\mathbf{N}d\xi, \tag{56}$$

$$\mathbf{m}_{\mathrm{eI}i} = \frac{\rho}{l}\int_{-1}^{1} \tilde{\mathbf{N}}^{\mathrm{T}}\boldsymbol{\eta}_i\boldsymbol{\eta}_i^{\mathrm{T}}\tilde{\mathbf{N}}d\xi, \tag{57}$$

$$\tilde{\mathbf{N}} = \int_{0}^{\xi} \mathbf{N}'^{\mathrm{T}}\mathbf{N}'d\xi. \tag{58}$$

And the linear stiffness matrix $\mathbf{k}_{\mathrm{eL}}$ and the geometrical nonlinear stiffness matrix $\mathbf{k}_{\mathrm{eG}i}$ for an element are

$$\mathbf{k}_{\mathrm{eL}} = \frac{EI}{l^3}\int_{-1}^{1} \mathbf{N}''^{\mathrm{T}}\mathbf{N}''d\xi, \tag{59}$$

$$\mathbf{k}_{\mathrm{eG}i} = \frac{EI}{l^5}\int_{-1}^{1} \mathbf{N}'^{\mathrm{T}}\mathbf{N}'\boldsymbol{\eta}_i\mathbf{N}''\boldsymbol{\eta}_i\mathbf{N}''d\xi \tag{60}$$

in which the subscript 'i' symbolizes the corresponding matrix of the ith element; ()'=$d/d\xi$; the superscript 'T' denotes the matrix transposition. These formulas indicate the nonlinear elemental matrices consist of quadratic terms and they are depends both upon the shape function and the nodal coordinates.

2. Nonlinear FEM of the entire NAM beam

With the elemental matrices of the coupled nonlinear oscillators and the nonlinear beam, the motion equation of the entire system can be formulated with a standard FEM procedure[5]. It is

$$\left[\mathbf{M}+\mathbf{M}_{\mathrm{I}}(\mathbf{q})\right]\ddot{\mathbf{q}}+\mathbf{C}\dot{\mathbf{q}}+\mathbf{K}_{\mathrm{L}}\mathbf{q}+\left[\mathbf{K}_{\mathrm{G}}(\mathbf{q})+\mathbf{K}_{\mathrm{r}}(\mathbf{q})\right]\mathbf{q} = \mathbf{F}(t) = \mathbf{F}\sin\omega t \tag{61}$$

where **M**, **K**, **C** are the mass, the stiffness and the damping matrices of the system, respectively. The subscripts, L, I, G, r, denote the variables corresponding to the linear case, nonlinear inertia, geometry and oscillator. $\mathbf{K}_{\mathrm{r}}(\mathbf{q})$ can be decoupled, but $\mathbf{M}_{\mathrm{I}}(\mathbf{q})$ and $\mathbf{K}_{\mathrm{G}}(\mathbf{q})$ are coupled.

We adopt the dynamic proportional damping model,

$$\mathbf{C} = c_0 \mathbf{K}_\mathrm{L} / \omega \tag{62}$$

where c_0 is a constant damping coefficient. In the main text and in this document, c_0=0.001 in the bifurcation analyses based on dimensional reduction method. In other method, the damping effect is neglect.

Method for frequency responses

1. Harmonic balance method (HBM)

The wave propagation depends on the periodic solutions. A frequency response is also a periodic solution. If the stability of a solution is unnecessary, HBM can be used. A first-order approximation of the solution is $\mathbf{q}(t) = \mathbf{Q}\sin\omega t$. Subscribing it into (61) and balancing the harmonic, we obtain the solution for the cubic nonlinear system,

$$\left[\mathbf{K}_\mathrm{L} - \omega^2\mathbf{M}\right]\mathbf{Q} + \frac{3}{4}\left[-\omega^2\mathbf{M}_\mathrm{I}(\mathbf{Q}) + \mathbf{K}_\mathrm{G}(\mathbf{Q}) + \mathbf{K}_\mathrm{r}(\mathbf{Q})\right]\mathbf{Q} = \mathbf{F}. \tag{63}$$

When the nonlinear matrices contain higher-order terms, the approach is still valid except for the different coefficients. However, there is larger round-off error for higher order nonlinear terms. In this case, we need to take into consideration of high-order harmonic waves, for example, supposing the solution is $\mathbf{q}(t) = \mathbf{Q}_1\sin\omega t + \mathbf{Q}_3\sin 3\omega t$.

The Newton iteration numerical algorithm is used to find the solutions of the vector $\mathbf{Q}$ with a specified force F at point E (see Fig. 2f,g). In this process, each cell of the NAM beam is meshed into two conformal elements, which is accurate in low-frequency range. Therefore, there are 78 DoFs in total for the NAM beam-N1 and the NAM beam-N2 has 90 DoFs.

However, HBM doesn't provide a regime to analyze the stability of a solution, so it cannot be directly used for bifurcation studies, even for simple cases. To reach this problem, we adopt the harmonic average approach (HAA) [6].

2. Harmonic average approach (HAA)

To analyze the stability of periodic solutions, we need to solve $[\mathbf{M} + \mathbf{M}_\mathrm{I}(\mathbf{q})]^{-1}$ to derive the Jacobian matrix of the system. However, it is difficult at present to solve a high-dimensional $[\mathbf{M}_\mathrm{I}(\mathbf{q})]^{-1}$ because it contains many unknown variables. Therefore we neglect the inertial nonlinearity in this method and our analyses will address that this neglecting does not introduce large errors.

We consider the equation

$$\mathbf{M}\ddot{\mathbf{q}} + \mathbf{C}\dot{\mathbf{q}} + \mathbf{K}_\mathrm{L}\mathbf{q} + \mathbf{k}_\mathrm{N}(\mathbf{q}) - \mathbf{F}(t) = 0 \tag{64}$$

where the vector $\mathbf{k}_\mathrm{N}(\mathbf{q}) = [\mathbf{K}_\mathrm{G}(\mathbf{q}) + \mathbf{K}_\mathrm{r}(\mathbf{q})]\mathbf{q}$. Within this approach, the solution is assumed to have the form:

$$\mathbf{q} = \mathbf{u}(t)\cos\theta + \mathbf{v}(t)\sin\theta, \tag{65}$$

$$\dot{\mathbf{q}} = -\omega\mathbf{u}(t)\sin\theta + \omega\mathbf{v}(t)\cos\theta, \tag{66}$$

where $\theta = \omega t$. The derivatives with respect to time t of the formulas in (65) and (66) are

$$\dot{\mathbf{q}} = (\dot{\mathbf{u}} + \omega\mathbf{v})\cos\theta + (\dot{\mathbf{v}} - \omega\mathbf{u})\sin\theta, \tag{67}$$

$$\ddot{\mathbf{q}} = (\omega\dot{\mathbf{v}} - \omega^2\mathbf{u})\cos\theta - (\omega\dot{\mathbf{u}} + \omega^2\mathbf{v})\sin\theta\,. \tag{68}$$

Comparing the expressions of $\dot{\mathbf{q}}$ in Eqs. (65) and (67), we obtain

$$\omega\mathbf{M}(\dot{\mathbf{u}}\cos\theta + \dot{\mathbf{v}}\sin\theta) = \mathbf{0} \tag{69}$$

The further substitution of (65) and (67) into (64) gives another form of the equation of motion:

$$\begin{gathered}\omega\mathbf{M}\left[\dot{\mathbf{v}}\cos\theta - \dot{\mathbf{u}}\sin\theta - \omega\mathbf{u}\cos\theta - \omega\mathbf{v}\sin\theta\right] + \omega\mathbf{C}\left[\mathbf{v}\cos\theta - \mathbf{u}\sin\theta\right] \\ +\mathbf{K}_{\mathrm{L}}[\mathbf{u}\cos\theta + \mathbf{v}\sin\theta] + \mathbf{k}_{\mathrm{N}}(\mathbf{u},\mathbf{v},\cos\theta,\sin\theta) - \mathbf{f}\sin\theta = 0\end{gathered}\,. \tag{70}$$

In practice, for the cubic nonlinear system, we can reduce the order of triangle functions in $\mathbf{k}_{\mathrm{N}}$ and rewrite it as

$$\mathbf{k}_{\mathrm{N}}(\mathbf{u},\mathbf{v},\cos\theta,\sin\theta) = \mathbf{k}_{\mathrm{N1}}(\mathbf{u},\mathbf{v})\cos\theta + \mathbf{k}_{\mathrm{N2}}(\mathbf{u},\mathbf{v})\cos 3\theta + \mathbf{k}_{\mathrm{N3}}(\mathbf{u},\mathbf{v})\sin\theta + \mathbf{k}_{\mathrm{N4}}(\mathbf{u},\mathbf{v})\sin 3\theta \tag{71}$$

Then, assuming that $\mathbf{u}$ and $\mathbf{v}$ are constant, by calculating (70)×sinθ-(69)×cosθ and integrating the result from 0 to 2π, we obtain

$$2\omega\mathbf{M}\dot{\mathbf{u}} = [\mathbf{K}_{\mathrm{L}} - \omega^2\mathbf{M}]\mathbf{v} - \omega\mathbf{C}\mathbf{u} + \mathbf{k}_{\mathrm{Nc}}(\mathbf{u},\mathbf{v}) - \mathbf{f}\,. \tag{72}$$

Similarly, calculating (70)×cosθ+(69)×sinθ and integrating over the interval [0, 2π] gives:

$$-2\omega\mathbf{M}\dot{\mathbf{v}} = [\mathbf{K}_{\mathrm{L}} - \omega^2\mathbf{M}]\mathbf{u} + \omega\mathbf{C}\mathbf{v} + \mathbf{k}_{\mathrm{Ns}}(\mathbf{u},\mathbf{v})\,. \tag{73}$$

The steady solutions correspond to the condition $\dot{\mathbf{u}} = \dot{\mathbf{v}} = \mathbf{0}$ and their expressions are

$$[\mathbf{K}_{\mathrm{L}} - \omega^2\mathbf{M}]\mathbf{v} - \omega\mathbf{C}\mathbf{u} + \mathbf{k}_{\mathrm{Nc}}(\mathbf{u},\mathbf{v}) = \mathbf{f}\,, \tag{74}$$

$$[\mathbf{K}_{\mathrm{L}} - \omega^2\mathbf{M}]\mathbf{u} + \omega\mathbf{C}\mathbf{v} + \mathbf{k}_{\mathrm{Ns}}(\mathbf{u},\mathbf{v}) = \mathbf{0}\,. \tag{75}$$

The amplitude of the response is $\mathbf{Y} = \sqrt{\mathbf{u}^2 + \mathbf{v}^2}$. Generally, we cannot obtain an analytical solution of these algebraic equations. Newton method and continuation are used to find the solution.

In fact, HBM can obtain the same result as (74), but with the differential formula, we can analyze the stability of a solution. We rewrite (72) and (73) to be

$$2\omega\dot{\mathbf{y}} = \mathbf{A}\mathbf{y} + R(\mathbf{y}) + \mathbf{F} \tag{76}$$

in which

$$\mathbf{y} = \begin{bmatrix}\mathbf{u}\\ \mathbf{v}\end{bmatrix}, \quad \mathbf{F} = \hat{\mathbf{M}}\begin{bmatrix}-\mathbf{f}\\ 0\end{bmatrix}, \tag{77}$$

$$\hat{\mathbf{M}} = \begin{bmatrix} \mathbf{M} & \\ & -\mathbf{M} \end{bmatrix}^{-1}, \tag{78}$$

$$\mathbf{A} = \hat{\mathbf{M}} \begin{bmatrix} -\omega\mathbf{C} & \mathbf{K}_{\mathrm{L}} - \omega^2\mathbf{M} \\ \mathbf{K}_{\mathrm{L}} - \omega^2\mathbf{M} & \omega\mathbf{C} \end{bmatrix}, \tag{79}$$

$$R(\mathbf{y}) = \hat{\mathbf{M}} \begin{bmatrix} \mathbf{k}_{\mathrm{Nc}}(\mathbf{u},\mathbf{v}) \\ \mathbf{k}_{\mathrm{Ns}}(\mathbf{u},\mathbf{v}) \end{bmatrix}. \tag{80}$$

Then the Jacobian matrix of the system described by (76) is

$$\mathbf{J} = \mathbf{A} + \frac{\partial R(\mathbf{y})}{\partial \mathbf{y}}. \tag{81}$$

The stability of a solution is determined by the real parts of the eigenvalues of $\mathbf{J}$: if there is an eigenvalue having a positive real part, the periodic solution is unstable; otherwise, it is stable.

However, though this stability theory of the periodic solution is rigorous in mathematics, for a high-dimensional system, it may provide wrong results because of numerical errors coming from the periodic solutions and the eigenvalues of $\mathbf{J}$. Moreover, the nonlinear dynamics developed at present is not enough to analyze the high-dimensional systems. To yield an accurate result, we need to reduce the dimensions of the original system. After the dimension-reduction, we can use HAA to solve the periodic solutions and implement a bifurcation analysis in frequency domain. The dimension-reduction is detailed in Supplementary Note 4.

Influences of nonlinear sources

The theoretical influences of different nonlinear sources on the NAM beam-N1 are illustrated in Supplementary Fig. 4. The influences of the vibro-impact oscillator are explained in Fig. 3c and not repeated here. As illustrated in Supplementary Fig. 4a, in the studied force range, the response of the geometrical-nonlinear beam is approximately equal to the geometrical & inertial-nonlinear beam in 0-760 Hz, which means that the inertial nonlinearity can be neglected if we had considered the geometrical nonlinearity in this situation. By comparing the linear beam and the geometrical-nonlinear beam, we find the nonlinear effect is broadband and the geometrical nonlinearity can help to suppress the infinite resonances especially in 0-20 Hz (because the beam generates larger deformations near the resonances in 0-20 Hz).

By comparing displacements at point A s_{A} under the three cases in Supplementary Fig. 4b, we find that: in 0-20 Hz (it is in 0-LR1), the geometrical nonlinearity plays a main effect; however, in the frequency range 20 Hz-LR2, especially near LR1, the Duffing oscillator plays a main effect to significantly suppress the elastic waves; the nonlinear effect deriving from the Duffing oscillator is broadband but it becomes weaker for the frequencies over LR2, meanwhile, the nonlinear effect from geometrical nonlinearity becomes a little stronger than the Duffing oscillator. Therefore in the

broadband analyses, the geometrical and inertial nonlinearities can be neglected when we had considered the nonlinear oscillators.

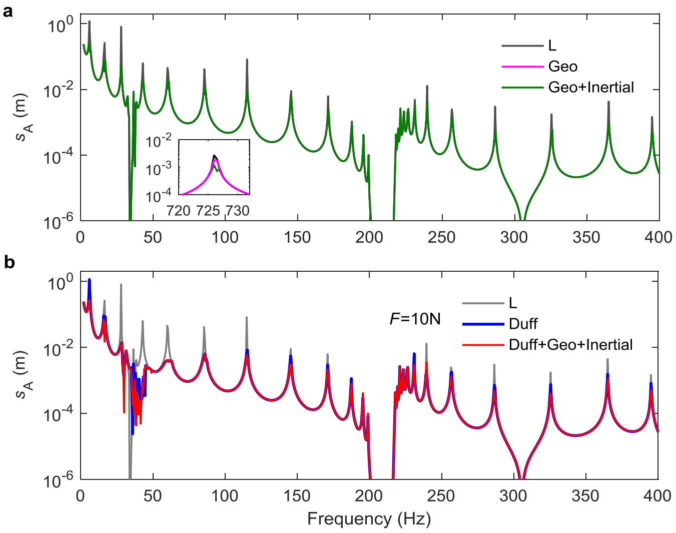

Supplementary Figure 4 | Influences of different nonlinear factors on NAM beam-N1. The displacement at point A s_A is present. The excitation force applied at point E is 10 N. Here a solution is solved by Newton method with a zero initial value. The dispersion properties are presented in Fig. 3b. 'L', 'Geo', 'Inertial' and 'Duff' represent linear, geometrical nonlinearity, inertial nonlinearity (shorten effect) and nonlinear Duffing oscillator cases, respectively. The vibro-impact oscillator is not considered here. (**a**) Three cases: linear; geometrical nonlinearity; geometrical & inertial nonlinearities. (**b**) Three cases: linear; Duffing oscillator; Duffing oscillator & geometrical & inertial nonlinearities.

Nonlinearity of the vibro-impact oscillator

In the theoretical description of the metamaterial beam and plate, the nonlinear restoring force of the vibro-impact oscillator is $P(x) = k_3 x + k_c x^n$, where $k_c = \alpha\delta^{-n}, \alpha \approx 1$. In Fig. 3, we analyses the nonlinear effect by taking n=3 under F=5 N as example in bifurcation analyses. In fact, we can obtain stronger nonlinear effect by increasing n. In Supplementary Fig. 5, we compare the broadband responses under F=2 N for two cases n=3 and n=5. Results indicate that the case n=5 obtains a broader effect that suppresses the elastic wave with a smaller excitation than the case n=3, especially in the third passband. Moreover, the responses near LR1 are approximately equal for the two cases. Therefore we can predict that, further increasing n will make the theoretical features in the frequency responses approach to the experimental results.

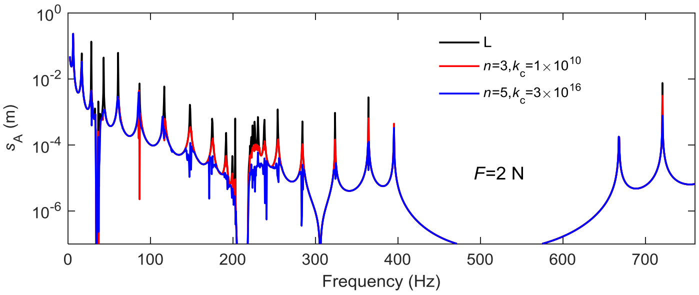

Supplementary Figure 5 | Different approximate orders of the vibro-impact oscillator. The excitation force F is 2 N. Here the solution is solved by Newton method with a zero initial value. The geometrical nonlinearity is not considered here. When n=3, k_c=1×10^{10}; when n=5, k_c=3×10^{16}.

Experiments on the LAM and NAM beams

In this section, an experiment on a LAM beam is introduced firstly. We remove all the magnets in the NAM cell in Fig. 2a to fabricate a cell of the LAM. There is a strut and a bolt left, which can be equivalent to a torsional local resonator that produces a LR bandgap. Influences of moderate deformation on the broadband frequency responses are measured.

For this LAM beam, under moderate deformations, there would be inertial and geometrical nonlinearities in the primary beam, but there is not nonlinearity came from the attached oscillators. As illustrated in Supplementary Fig. 6, the transfer functions under two remarkably different excitations remain nearly constant in 0-2000 Hz, except for from some local small differences. Those local differences are deemed to be introduced by the geometrical nonlinearity and the unavoidable system errors. This experiment proves that: the experimental system is robust and the noise-signal ratio is acceptable; the influences of inertial and geometrical nonlinearities on the acoustic metamaterial beam are not apparent under the excitation levels in our experiments, which demonstrates the results in theoretical analyses.

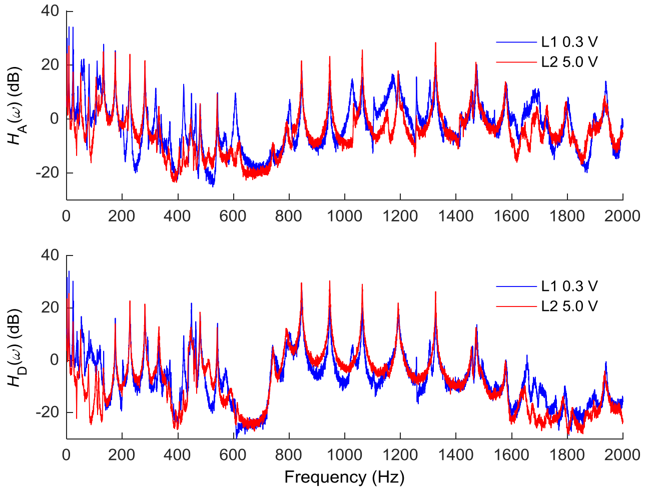

Supplementary Figure 6 | Transfer functions of the LAM beam under small and moderate deformations. $H_A(\omega)$ and $H_D(\omega)$ symbolize the transfer functions at points A and D, respectively. D is the center point between A and B on the beam (see Fig. 2f). L1: the excitation

voltage of the amplifier is 0.3 V and the average driving displacements in the interval (0, 20 Hz) is 5.643×10^{-4} mm; L2: the voltage is 5.0 V (5.065×10^{-2} mm). If the averages are made in the interval (0, 2000 Hz), the average driving amplitude also increases 61.9 times from 0.3 V to 5.0 V.

Secondly, we will directly compare the responses of the LAM beam and the NAM beam. As well established, the frequency response of a linear system increases linearly with the excitation, therefore we can derive its response under a higher excitation from the response under a small excitation, and vice versa. Among the experiments on the NAM beam shown in Fig. 3a, case i 0.1 V is a linear case but case iv 5.8 V is a strongly nonlinear case. By comparing the averaged excitation velocities $\bar{v}_e$ of the two cases, we get $\bar{v}_{e,iv} = 64.2\bar{v}_{e,i}$ in a broad band. Indeed, as illustrated in Supplementary Fig. 7, for the spectra of the excitation velocities $v_e(f)$, we have $v_{e,iv}(f) = 64.2v_{e,i}(f)$ though there are tiny local differences, where f denotes the frequency. This result demonstrates further the robustness of the experimental apparatus. Therefore $64.2v_{rsp,i}$ should be the response of the linearized system in case iv (it is a supposed value but not a true value), that is, $v_{rsp,\,iv,L} = 64.2v_{rsp,\,i}$, where the subscript 'L' symbolizes 'linearized'. $v_{rsp,\,iv}$ is the true response in case iv. By comparing $v_{rsp,\,iv,L}$ with $v_{rsp,\,iv}$, we obtain the influences of the nonlinearities in the NAM beam on the wave propagation. The behaviors shown by the responses are identical as the transfer functions in Fig. 3a: wave propagation in NAM beam is suppressed in an ultra-low and ultra-broad band.

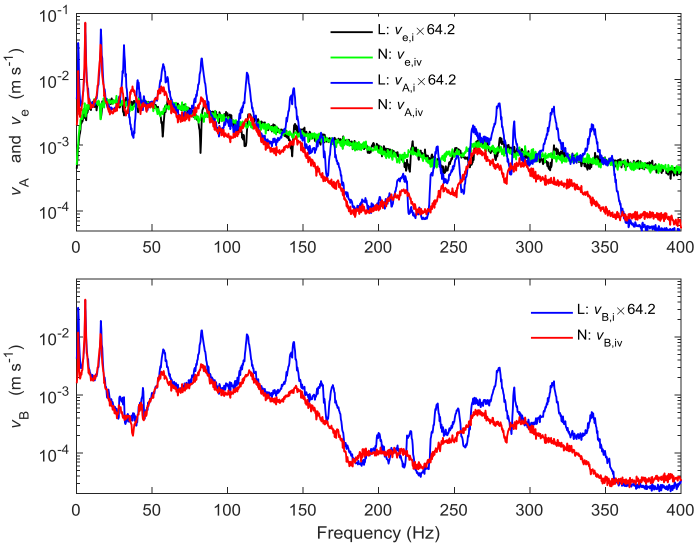

Supplementary Figure 7 | Response velocities of the NAM beam under two excitations. Here, v_{rsp} at points A and B are symbolized by v_A and v_B, respectively. N: case iv 5.8 V in Fig. 3; L: the linearized case iv derived from case i 0.1 V in Fig. 3; Different cases are indicated in the legends.

Supplementary note 4. Dimension-reduction method

Bifurcation properties can help to directly demonstrate the chaotic mechanism in theory. For a low-dimensional model,

HAA can present bifurcations of periodic solutions accurately[6]. However, for the high-dimensional model of the NAM beam, HAA would give wrong stability information because of the numerical errors coming from the periodic solutions and the eigenvalues of the Jacobian matrix (eigenvalues are sensitive to periodic solutions). To analyze the bifurcations of the periodic solutions of the NAM beam, we need practical dimension-reduction algorithm[7] in frequency domain to reduce the dimension of the finite element model.

Here we adopt the thought of the post-processed Galerkin method[8] in frequency domain to reduce the dimension of the NAM beam and then we analyze the periodic solutions and their bifurcations based upon the reduced system.

Dimension-reduction algorithm

The dimension d of a system is the number of the second-order differential equations (64). We adopt a rigorous and practical dimension-reduction approach based on the orthogonality of linear modals.

The eigenvalues of the matrix $\mathbf{B} = \mathbf{M}^{-1}\mathbf{K}_\mathrm{L}$ are $\mu_j = \omega_j^2$, and the corresponding eigenvector is $\boldsymbol{\varphi}_j$, that is, $\mathbf{B}\boldsymbol{\varphi}_j - \mu_j \boldsymbol{\varphi}_j = \mathbf{0}$. ω_j is the jth-order nature frequency. Based on the modal orthogonality, we have

$$\boldsymbol{\varphi}_j^\mathrm{T}[\mathbf{X}]\boldsymbol{\varphi}_i = \begin{cases} 0, & i \neq j \\ h_j, & i = j \end{cases}, \tag{82}$$

in which the matrix [**X**] represents **M** or $\mathbf{K}_\mathrm{L}$, the corresponding h_j is named as ‘modal mass’ or ‘modal stiffness’. Generally, for the damping matrix, $\boldsymbol{\varphi}_j^\mathrm{T}\mathbf{C}\boldsymbol{\varphi}_i \neq 0$ for $i \neq j$. But if we adopt the dynamic proportional damping model in Eq. (62), $\boldsymbol{\varphi}_j^\mathrm{T}\mathbf{C}\boldsymbol{\varphi}_i = 0$ for $i \neq j$.

To reduce the dimension, firstly, let’s sort the eigenvalues as $|\mu_1| \le |\mu_2| \le |\mu_3| \cdots$. And the corresponding eigenvectors are $\varphi_1, \varphi_2, \cdots$. For a solution **q** we make the ansatz

$$\mathbf{q} = \mathbf{H}_k\mathbf{x} + \mathbf{P}_k\mathbf{y}, \tag{83}$$

where the columns of the matrix $\mathbf{H}_k = [\varphi_1, \varphi_2, \cdots, \varphi_k]$ span the k-dimensional sub-space $H_k = \mathrm{span}\{\varphi_1, \varphi_2, \cdots, \varphi_k\}$ formed by the first k eigenvectors. Similarly, $P_k = \mathrm{span}\{\varphi_{k+1}, \varphi_{k+2}, \cdots, \varphi_n\}$ and $\mathbf{P}_k = [\varphi_{k+1}, \varphi_{k+2}, \cdots, \varphi_n]$.

Secondly, subscribing (83) in (64) and specifying the nonlinear operator $R(\mathbf{q}) = \mathbf{k}_\mathrm{N}(\mathbf{q})$ yield

$$\mathbf{M}(\mathbf{H}_k\ddot{\mathbf{x}} + \mathbf{P}_k\ddot{\mathbf{y}}) + \mathbf{C}(\mathbf{H}_k\dot{\mathbf{x}} + \mathbf{P}_k\dot{\mathbf{y}}) + \mathbf{K}_\mathrm{L}(\mathbf{H}_k\mathbf{x} + \mathbf{P}_k\mathbf{y}) + R(\mathbf{x}, \mathbf{y}) = \mathbf{F}(t). \tag{84}$$

By left multiplying $\mathbf{H}_k^\mathrm{T}$ and $\mathbf{P}_k^\mathrm{T}$ by Eq. (84) respectively, we obtain

$$\mathbf{M}_1\ddot{\mathbf{x}} + \mathbf{C}_1\dot{\mathbf{x}} + \mathbf{K}_1\mathbf{x} + \mathbf{H}_k^\mathrm{T}R(\mathbf{H}_k\mathbf{x} + \mathbf{P}_k\mathbf{y}) = \mathbf{H}_k^\mathrm{T}\mathbf{F}(t), \tag{85}$$

$$\mathbf{M}_2\ddot{\mathbf{y}} + \mathbf{C}_2\dot{\mathbf{y}} + \mathbf{K}_2\mathbf{y} + \mathbf{P}_k^\mathrm{T}R(\mathbf{H}_k\mathbf{x} + \mathbf{P}_k\mathbf{y}) = \mathbf{P}_k^\mathrm{T}\mathbf{F}(t), \tag{86}$$

where $\mathbf{M}_1 = \mathbf{H}_k^\mathrm{T}\mathbf{M}\mathbf{H}_k$, $\mathbf{C}_1 = \mathbf{H}_k^\mathrm{T}\mathbf{C}\mathbf{H}_k$, $\mathbf{K}_1 = \mathbf{H}_k^\mathrm{T}\mathbf{K}_\mathrm{L}\mathbf{H}_k$, $\mathbf{M}_2 = \mathbf{P}_k^\mathrm{T}\mathbf{M}\mathbf{P}_k$, $\mathbf{C}_2 = \mathbf{P}_k^\mathrm{T}\mathbf{C}\mathbf{P}_k$, $\mathbf{K}_2 = \mathbf{P}_k^\mathrm{T}\mathbf{K}_\mathrm{L}\mathbf{P}_k$.

The first formula in (85) is the reduced system. Then, the problem comes to be how to solve the system. The traditional

Galerkin approach neglect the influences of high-order modals by specifying $\mathbf{y}$=0, so the reduced system is

$$\mathbf{M}_1\ddot{\mathbf{x}}+\mathbf{C}_1\dot{\mathbf{x}}+\mathbf{K}_1\mathbf{x}+\mathbf{H}_k^{\mathrm{T}}R(\mathbf{H}_k\mathbf{x})=\mathbf{H}_k^{\mathrm{T}}\mathbf{F}(t). \tag{87}$$

Based on the approximate inertial manifold theory, the nonlinear Galerkin method takes into consideration of the effects of the higher eigenmodes. In this method, we need to formulate a graph ($\mathbf{x}$, Φ_{app}) that makes $\mathbf{y}=\Phi_{\mathrm{app}}(\mathbf{x})$. By specifying $\ddot{\mathbf{y}}=0, \dot{\mathbf{y}}=0$ in the second formula of (85), one yields

$$\Phi_{\mathrm{app}}(\mathbf{x})=\mathbf{K}_2^{-1}\left\{-\mathbf{P}_k^{\mathrm{T}}R(\mathbf{H}_k\mathbf{x}+\mathbf{P}_k\mathbf{y})+\mathbf{P}_k^{\mathrm{T}}\mathbf{F}(t)\right\}. \tag{88}$$

In practice, Picard iterations are used to solve $\Phi_{\mathrm{app}}(\mathbf{x})$, for example,

$$\Phi_1(\mathbf{x})=\mathbf{K}_2^{-1}\left\{-\mathbf{P}_k^{\mathrm{T}}R(\mathbf{H}_k\mathbf{x})+\mathbf{P}_k^{\mathrm{T}}\mathbf{F}(t)\right\}, \tag{89}$$

$$\Phi_2(\mathbf{x})=\mathbf{K}_2^{-1}\left\{-\mathbf{P}_k^{\mathrm{T}}R(\mathbf{H}_k\mathbf{x}+\mathbf{P}_k\Phi_1(\mathbf{x}))+\mathbf{P}_k^{\mathrm{T}}\mathbf{F}(t)\right\}. \tag{90}$$

Therefore, the reduced system in this method is

$$\mathbf{M}_1\ddot{\mathbf{x}}+\mathbf{C}_1\dot{\mathbf{x}}+\mathbf{K}_1\mathbf{x}+\mathbf{H}_k^{\mathrm{T}}R(\mathbf{H}_k\mathbf{x}+\mathbf{P}_k\Phi_{\mathrm{app}}(\mathbf{x}))=\mathbf{H}_k^{\mathrm{T}}\mathbf{F}(t)\ . \tag{91}$$

However, it is time consuming to solve $\Phi_{\mathrm{app}}(\mathbf{x})$ in frequency domain because the nonlinear operator $R(\mathbf{x},\mathbf{y})$ needs many high-order symbol calculations. For example, if the original nonlinear operator is cubic nonlinearity, $\Phi_2(\mathbf{x})$ becomes nine-order and then Eq. (91) becomes 27-order.

This paper adopts the idea of the post-processed Galerkin thought. The steps of this algorithm are listed below.

Step 1: Solve Eq. (87) with HAA by the ansatz $\mathbf{x}=\mathbf{a}\cos\omega t+\mathbf{b}\sin\omega t$, it is

$$(\mathbf{K}_1-\omega^2\mathbf{M}_1)\mathbf{b}-\omega\mathbf{C}_1\mathbf{a}+\mathbf{H}_kR_{\mathrm{s}}(\mathbf{a},\mathbf{b})=0, \tag{92}$$

$$(\mathbf{K}_1-\omega^2\mathbf{M}_1)\mathbf{a}+\omega\mathbf{C}_1\mathbf{b}+\mathbf{H}_kR_{\mathrm{c}}(\mathbf{a},\mathbf{b})=\mathbf{H}_k\mathbf{f}, \tag{93}$$

where $R(\mathbf{H}_k\mathbf{x})=R_{\mathrm{s}}(\mathbf{a},\mathbf{b})\sin\omega t+R_{\mathrm{c}}(\mathbf{a},\mathbf{b})\cos\omega t+o(\sin 3\omega t, \cos 3\omega t)$, in which $o(\cdot)$ denotes the third-order value.

Step 2: Determine the stability of the reduced system by Jacobian matrix

$$J_1=\mathbf{A}_1+\frac{\partial\hat{R}(\mathbf{a},\mathbf{b})}{\partial\mathbf{X}} \tag{94}$$

where $\mathbf{X}=[\mathbf{a}^{\mathrm{T}}\quad \mathbf{b}^{\mathrm{T}}]^{\mathrm{T}}$ and

$$\hat{\mathbf{M}}_1=\begin{bmatrix}\mathbf{M}_1 & \\ & -\mathbf{M}_1\end{bmatrix}^{-1}, \tag{95}$$

$$\mathbf{A}_1=\hat{\mathbf{M}}_1\begin{bmatrix}-\omega\mathbf{C}_1 & \mathbf{K}_1-\omega^2\mathbf{M}_1\\ \mathbf{K}_1-\omega^2\mathbf{M}_1 & \omega\mathbf{C}_1\end{bmatrix}, \tag{96}$$

$$\hat{R}(\mathbf{a},\mathbf{b}) = \hat{\mathbf{M}}\begin{bmatrix}\mathbf{H}_k R_s(\mathbf{a},\mathbf{b})\\ \mathbf{H}_k R_c(\mathbf{a},\mathbf{b})\end{bmatrix}. \tag{97}$$

The stabilities of the periodic solutions of the original system are same with that of the reduced system.

Step 3: With the solved $\mathbf{x}$, we post-process that $\mathbf{y} = \Phi_1(\mathbf{x}) = \Phi_1(\mathbf{a}\cos\omega t + \mathbf{b}\sin\omega t)$ and adopt the first-order harmonic approximation.

Step 4: The periodic solution of the original system is solved with Eq. (83).

Bifurcations in the NAM beam-N1

To demonstrate the validity of the dimension-reduction algorithm, we reduce the dimension of the finite element model of NAM beam-N1 from 78 to 20 to study the bifurcations of periodic solutions, as illustrated in Supplementary Fig. 8. Geometrical nonlinearity is considered here. In the dimension-reduction procedure, we consider the first 20 linear eigenmodes to approximate the responses in 0-120 Hz. In the next section, we will introduce how to expand this frequency range. Moreover, perturbation continuation methom[6] is used to find different branches of solutions. Considering the numerical errors, we used a tiny eigenvalue μ_c=1×10^{-8} of the Jacobian matrix as the critical value to determine the stability of a solution. In fact, for unstable solutions, the positive real parts of the eigenvalues of Jacobian matrix can be very large; in contrast, they are negative or just in the magnitude 10^{-12} for stable solutions. In Fig. 3, we also use this threshold value for NAM beam-N2.

As suggested in Supplementary Fig. 8, near 16 Hz, because the existence of cubic geometrical nonlinearity, the nonlinear resonance consists of 3 branches bending right, among which two branches are stable and another branch is unstable, which is like the nonlinear resonance of a 1DoF nonlinear system. This nonlinear resonance demonstrates the accuracy of the dimension-reduction algorithm. In contrast, near 60 Hz and 85 Hz, nonlinear resonances consist of unstable branches or unstable peaks whose amplitudes are much lower than the linearized responses. Those unstable branches provide high possibilities of chaotic responses, as well established in Ref. [6].

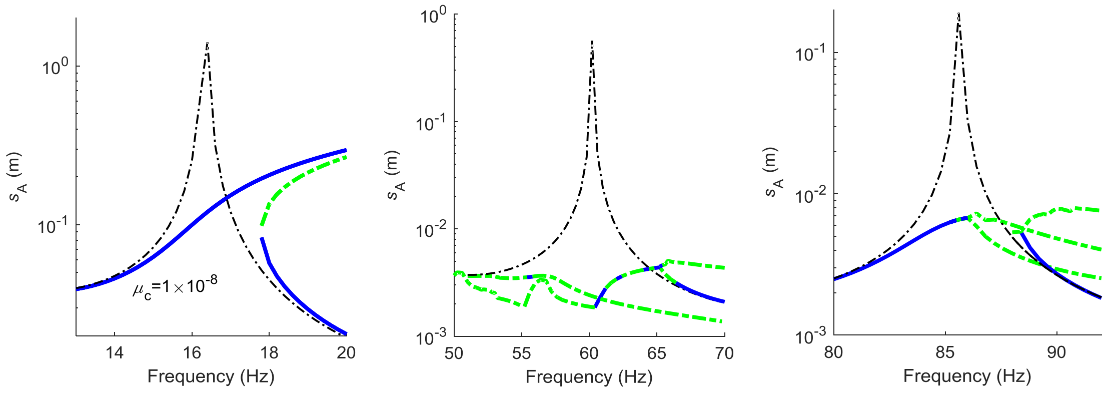

Supplementary Figure 8 | Bifurcations of the periodic solutions of the reduced system NAM beam-N1. The constant force *F*=20N. Thin black lines: solutions of LAM; solid blue (dashed green): stable (unstable) periodic solutions of the NAM.

Validity of the dimension-reduced method for NAM beam-N2

There are 90 dimensions for the NAM beam-N2. Here, the geometrical nonlinearity can be neglected because of the strongly nonlinearity coming from the vibro-impact oscillators. However, because the NAM beam consists of periodic nonlinear local resonators, there are dense LR eigenmodes whose frequencies are approximately equal. For example, the first 37 eigen-frequencies of the linearized NAM beam-N2 are (in descending order):

202.601, 199.27, 191.445, **174.784, 147.76, 116.412, 86.3573, 60.5525, 42.9743, 38.1879, 37.6735, 36.4054, 36.3972, 33.6655, 33.6534**, 33.6279, 33.5766, 33.467, **33.1973, 32.3269, 28.0274, 16.3884, 10.671**, 10.6602, 10.6579, 10.6579, 10.6578, 10.6577, 10.6576, 10.6574, 10.6571, **10.6564, 10.6547, 10.6453**, **6.10696, 0.000073698*i*, 0.000015911**

As shown in this list, the two teams of underlined frequencies are approximately equal. If we want to cover the responses in 0-175 Hz in the reduced system, the original dimension-reduction approach has to consider 34 eignenmodes, which is so high that the calculation needs a very long time. To reduce the dimension more but still keep the accuracy in 0-175 Hz, actually we can pick the 23 eigenmodes whose frequencies are thickened in the list. We name this procedure as picking dimension-reduction approach. The picking method can filter the approximately equal local-resonant eigenmodes.

As shown in Supplementary Fig. 9, the response of the reduced system is nearly equal to the original un-reduced system in this frequency range except for a small difference near 85 Hz, which demonstrate the dimension-reduction algorithm is valid.

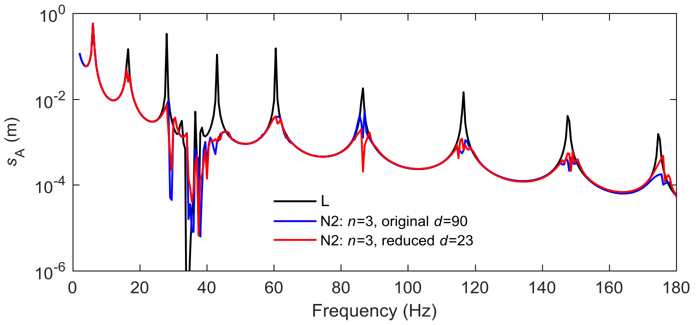

Supplementary Figure 9 | Comparison of the dimension-reduced system and the original system. In the nonlinear FEM, the original system has 90 dimensions. The reduced system has 23 dimensions which cover the responses in 0-175 Hz. *F*=5 N.

Supplementary References

1. Narisetti, R. K., Ruzzene, M. & Leamy, M. J. A perturbation approach for analyzing dispersion and group velocities in two-dimensional nonlinear periodic lattices. *J. Vib. Acoust.* **133**, 061020 (2011).

2. Fang, X., Wen, J., Yin, J. & Yu, D. Wave propagation in nonlinear metamaterial multi-atomic chains based on homotopy method.

AIP Adv. **6**, 121706 (2016).

3. Wriggers, P. *Nonlinear finite element methods.* (Springer, 2008).

4. Me, C. & Decha-Umphai, K. A finite element method for non-linear forced vibration of beams. *J. Sound Vib.* **102** (3), 369-380 (1985)

5. Petyt, M. *Introduction to finite element vibration analysis, 2nd edition.* (Cambridge University Press, 2010).

6. Fang, X., Wen, J., Bonello, B., Yin, J. & Yu D. Bifurcations and chaos in nonlinear acoustic metamaterials. *New J. Phys.* **19**, 053007 (2017).

7. Rega, G. & Troger, H. Dimension reduction of dynamical systems: Methods, models, applications. *Nonlinear Dyn.* **41**, 1–15 (2005).

8. Laing, C. R., McRobie, A. & Thompson, J. M. The post-processed Galerkin method applied to non-linear shell vibrations. *Dynamics and Stability of Systems*, **14** (2), (1999).
